# Characterization and biodistribution of under-employed gene therapy vector AAV7

**DOI:** 10.1128/jvi.01163-23

**Published:** 2023-10-16

**Authors:** Samantha A. Yost, Emre Firlar, Justin D. Glenn, Hayley B. Carroll, Steven Foltz, April R. Giles, Jenny M. Egley, Elad Firnberg, Sungyeon Cho, Trang Nguyen, William M. Henry, Karolina J. Janczura, Joseph Bruder, Ye Liu, Olivier Danos, Subha Karumuthil-Melethil, Sanjana Pannem, Valerie Yost, Yelena Engelson, Jason T. Kaelber, Hemi Dimant, Jared B. Smith, Andrew C. Mercer

**Affiliations:** 1 Research and Early Development, REGENXBIO Inc., Rockville, Maryland, USA; 2 Institute of Quantitative Biomedicine and Rutgers CryoEM & Nanoimaging Facility, Rutgers, The State University of New Jersey, Piscataway, New Jersey, USA; 3 Invicro LLC, Needham, Massachusetts, USA; 4 Emit Imaging, Baltimore, Maryland, USA; Cornell University Baker Institute for Animal Health, Ithaca, New York, USA

**Keywords:** gene therapy, AAV, adeno-associated vectors, biodistribution, cryoelectron microscopy, virology

## Abstract

AAV7 is a clade D adeno-associated virus (AAV) whose potential as a gene therapy vector has not been fully evaluated. We show here that high-titer AAV7 vectors can be produced in HEK293 cells and used to transduce cultured cells in an AAV receptor (AAVR)-dependent manner. In contrast with AAV9, AAV7 did not significantly interact with glycans when assayed on a panel of three hundred biologically relevant species. The structure of the AAV7 capsid was obtained by single particle cryo-electron microscopy at 2.7 Å, showing the expected AAV scaffold as well as unique features in variable surface loops. The biodistribution profiles of AAV7 and AAV9 vectors were compared in mice following intravenous administration and quantification of vector genomes and mRNA in tissues, as well as cryofluorescence tomography (CFT) and immunofluorescence (IF). AAV7 displayed a strong cardiac tropism and did not efficiently cross the blood-brain barrier. Surprisingly, strong transgene expression was observed throughout the head as shown by CFT, including regions of the cranial sinuses, teeth, and mandible for both AAV7 and AAV9, an attribute that has not been previously evaluated via traditional biodistribution methods.

**IMPORTANCE:**

The use of adeno-associated viruses (AAVs) as gene delivery vectors has vast potential for the treatment of many severe human diseases. Over one hundred naturally existing AAV capsid variants have been described and classified into phylogenetic clades based on their sequences. AAV8, AAV9, AAVrh.10, and other intensively studied capsids have been propelled into pre-clinical and clinical use, and more recently, marketed products; however, less-studied capsids may also have desirable properties (e.g., potency differences, tissue tropism, reduced immunogenicity, etc.) that have yet to be thoroughly described. These data will help build a broader structure-function knowledge base in the field, present capsid engineering opportunities, and enable the use of novel capsids with unique properties.

## INTRODUCTION

Adeno-associated viruses (AAVs) are a group of non-pathogenic, DNA viruses of the *Dependoparvovirus* genus and *Parvoviridae* family. These small, non-enveloped viruses capable of transducing both dividing and quiescent cells are currently being used clinically and pre-clinically in the field of gene therapy (1, 2). Although their safety profile and ability to deliver genetic cargo to cells are promising, further engineering opportunities exist in order to improve tissue specificity, immune detection avoidance, and ease of manufacturing (3). The 25 nm icosahedral AAV is composed of 60 subunits of viral capsid protein surrounding a packaged genome of approximately 4.7 kb (4). Over one hundred naturally occurring AAV serotypes and variants with differing properties have been described in the literature (5). The overall capsid protein structure of all serotypes described by X-ray crystallography and/or cryo-electron microscopy unsurprisingly contain the same overall fold, suggesting that biochemical differences are mainly attributed to sequence diversity located in several variable region (VR) loops on the capsid surface or to the flexible N-terminus (6). AAV isolates have been organized into clades (A through F) according to their sequence similarity (7).

AAV7 is in clade D, a group containing several understudied capsids initially found in rhesus or cynomolgus macaque tissue (8). Its capsid protein (VP1) has an 87.8%, 81.6%, and 88.4% identity of amino acids compared to widely used serotypes AAV8, AAV9, and AAVrh.10, respectively. Literature examining AAV7 thus far has been incomplete, lacking direct comparison to other, and more widely described AAVs. When injected into the cisterna magna of macaques, AAV7 vectors exhibit widespread transgene expression in both neurons and astrocytes in the brain and spinal cord (9). Comparison of enhanced green fluorescent protein (EGFP) expression in transduced tissue suggests better performance by AAV7 versus AAV9, the current gold standard for central nervous system (CNS) transduction; however, the two EGFP-expression constructs in this study were not driven by identical promoters (chicken β-actin promoter and cytomegalovirus promoter for AAV7 and AAV9, respectively), making protein expression results incomparable (10). In a mouse xenograft liver model, intravenous (IV) administration of AAV7 showed transduction of human hepatocytes at a similar level to AAV8 (11). Taken together with the evidence that AAV7 is readily produced in HEK293 systems (12), AAV7 could be a prime choice as a gene therapy vector candidate.

To further establish AAV7 as a viable gene therapy capsid for clinical use, we sought to fully characterize this serotype through biochemical and structural examination, and several methods of *in vivo* biodistribution experimentation.

## RESULTS AND DISCUSSION

### Production and *in vitro* transduction

AAV7, AAV8, and AAV9 particles were produced via triple transfection of HEK293 cells. Statistical analysis found no significant difference in production of AAV7, AAV8, and AAV9 produced by suspension-adapted HEK293 cells secreted in the supernatant 5 days post-transfection (>10^10^ GC/mL of culture) or adherent HEK293 cells 3 days post-transfection after lysis and iodixanol purification (>10^13^ GC/mL), supporting previous reports (12). Packaging efficiency of AAV7 is therefore comparable to AAV8 and AAV9 and is suitable for manufacturing.

**Fig 1:**
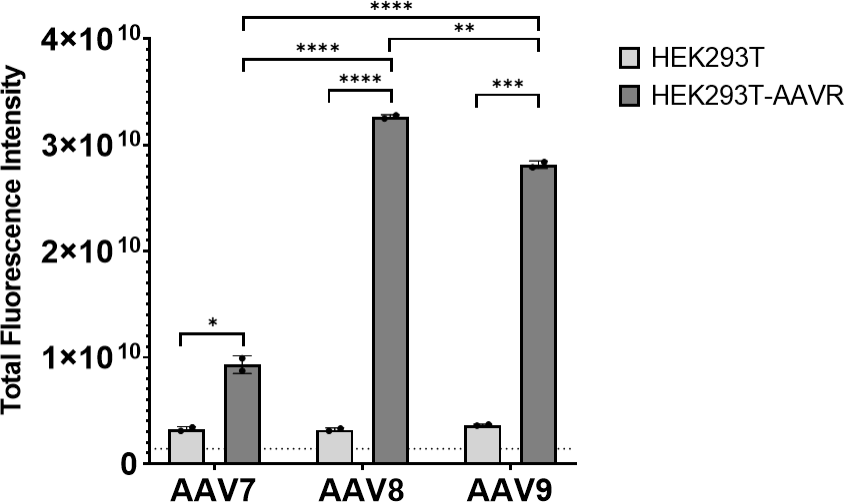
Total fluorescence intensity of HEK293T or HEK203T-AAVR cells 2 days post-transduction with 6.25 × 105 GC/cell CAG.tdTomato expression AAVs (*n* = 2). *P ≤ 0.05, **P ≤ 0.01, ***P ≤ 0.001, ****P ≤ 0.0001.

There has been some discrepancy in the literature regarding AAV7 dependence on AAV receptor (AAVR) (13). AAVR knock-out of U-2 OS cells severely impaired AAV7 transduction (14), but over-expression of AAVR in HEK293 subclone 84–31 had no significant effect on transduction at an MOI of 5 × 10^5^ (15). In this study, iodixanol-purified vector was used to transduce HEK293T and HEK293T-AAVR cells to evaluate *in vitro* transduction efficiency. AAV7 was able to transduce HEK293T cells at similar levels compared to AAV8 and AAV9 based on total TdTomato fluorescence; however, in HEK293T-AAVR cells, AAV9 and AAV8 transduction is significantly greater than AAV7 (Fig. 1). Direct comparison of AAV7 transduction between cell lines shows a 2.8-fold increase in transduction in the presence of AAVR overexpression (Fig. 1). Based on sequence and structural similarities, we hypothesize that AAV7 may interact with the polycystic kidney disease repeat domain 2 (PKD2) of AAVR at the 2/5-fold wall as seen with AAV1, AAV2, and AAV9 (16 – 18). T273 is a notable change in the predicted AAVR binding surface of AAV7 as compared to AAV2 (H271) and AAV9 (A273) that could contribute to a reduction in binding affinity.

Screening of AAV7 receptor binding through a cell-based array of membrane proteins did not produce any meaningful hits, even though AAV receptor (AAVR) was included (data not shown). Taken together, this data may imply a weaker binding interaction between AAV7 and AAVR, compared to AAV8 and AAV9, and/or an additional dependence on glycans, GPR108 (14), or other binding factors. If AAV7 must interact with both AAVR and glycans or GPR108, either simultaneously or sequentially, transduction will be impaired with the reduction of AAVR, but not increased beyond the level of glycan/GPR108 availability when AAVR is overexpressed (i.e., glycan/GPR108 becomes the limiting factor of transduction). In contrast, for AAV8 and AAV9, it appears that overexpression of AAVR alone is sufficient for increasing transduction by 10-fold in this study as well as in previously published work (15).

### Glycan binding analysis

**Fig 2:**
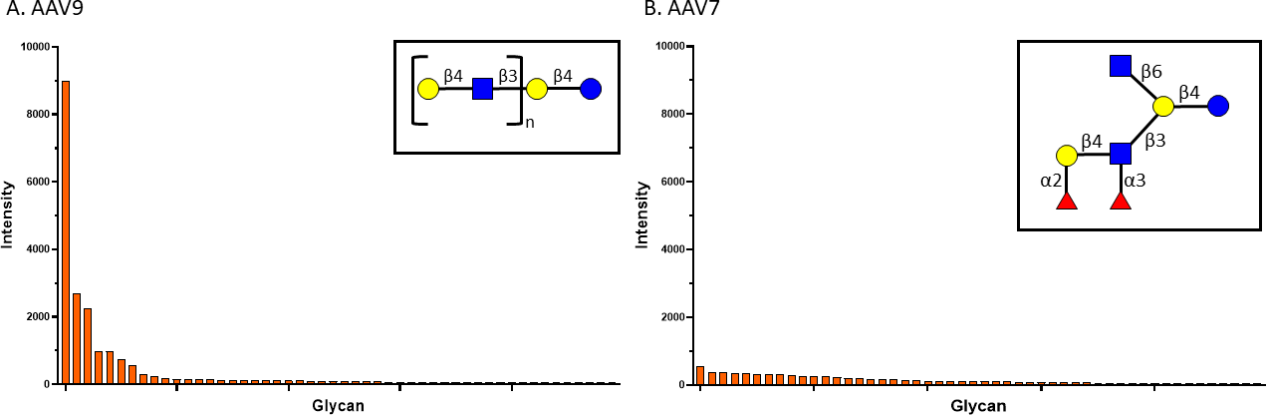
Top 50 glycan array hits sorted by intensity for AAV9 (**A**) and AAV7 (**B**). (**A**) A common glycan backbone found to be enriched in the top AAV9 hits is shown in the inset panel where “N” represents a variably repeated two-residue subunit. (**B**) The top AAV7-binding glycan is shown in the inset panel.

Glycan receptor identification for AAV7 previously failed (19); therefore, we performed a broad glycan-binding assay containing 300 biologically relevant glycans in array. AAV9 binds preferentially to glycans containing N-terminal galactose residues (20), thus we used AAV9 as a positive control (Fig. 2A). Glycan array analysis confirmed preferential binding of N-terminal galactose by AAV9: in the top 30 glycans bound by AAV9, glycans containing an N-terminal galactose and a common backbone were found to be enriched (Fig. 2A). Glycan array analysis of AAV7 shows a much more indiscriminate binding profile compared to AAV9 (Fig. 2B). N-terminal residues and branching patterns are variable among the AAV7-binding glycans. Of the top 10 binding partners, four are fucosylated human milk oligosaccharides, the top one being Fuc-α−1,2-Gal-β−1,4-(Fuc-α−1,3-)GlcNAc-β−1,3-(GlcNAc-β−1,6-)Gal-β−1,4-Glc-Sp5; however, these interactions may be non-specific as human milk oligosaccharides are known to have general antimicrobial and antiviral effects (21). Further characterization of the AAV7-glycan interaction has been hindered thus far due to the complexity of glycan synthesis.

### Cryo-EM structure

**Fig 3:**
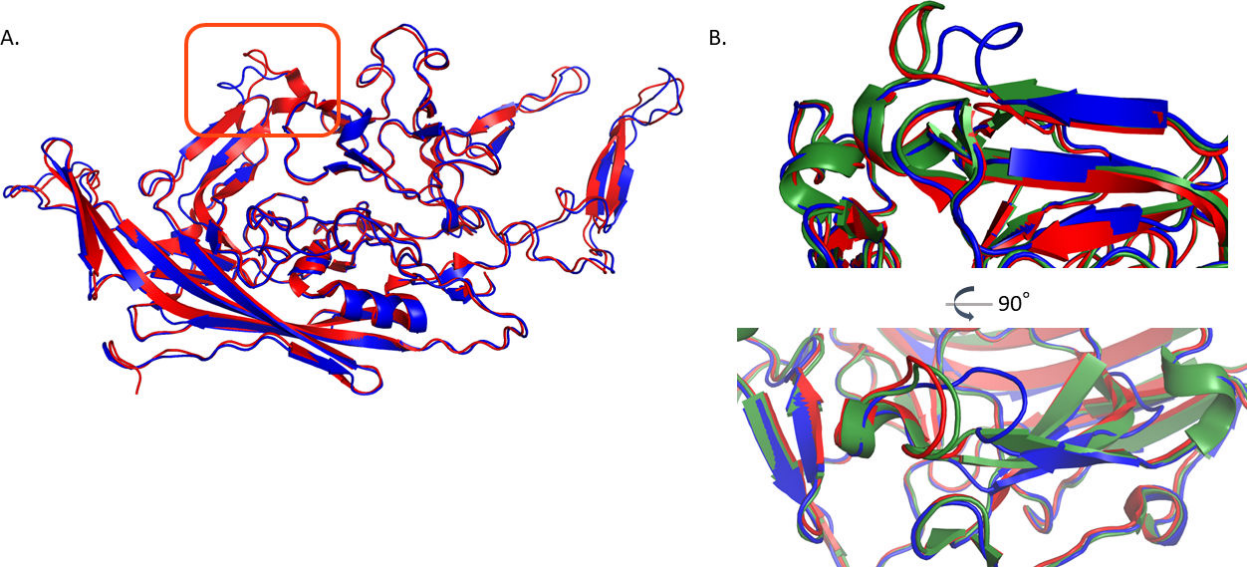
Atomic structure of AAV7 capsid protein VP3. (**A**) Cartoon representation of AAV7 (blue) and AAVrh.10 (red; PDBID: 6V1G). The main backbone differences lie in VR-I (boxed in orange; RMSD 1.85 Å). (**B**) Zoomed in representation of VR-I loop as colored in panel A with the addition of AAV8 (green; PDBID: 6V10) in two views.

Toward fully characterizing the biophysical properties of AAV7, we subjected genome-containing AAV7 particles to cryo-EM single-particle analysis (PDBID: 7JOT, EMDB: EMD-22412). We resolved the icosahedrally-averaged structure to 2.7 Å resolution map and derived a VP3 structural model (Table 1). AAV7 capsid structure contains the typical eight antiparallel β-strands with an α-helix core common across all described AAV VP3s (Fig. 3). Since the collection and deposition of our structural data, two additional AAV7 structures have been deposited in online databases: one genome-containing particle (PDBID: 7L5U) and one empty particle (PDBID: 7L5Q) (22). The two genome-containing structures do not differ in any significant way supported by density; although the root mean square deviation (RMSD) is 0.7 Å, this is mostly attributable to differences in the pixel size calibration causing isotropic stretching of one model with respect to the other. At 2.7 Å, the HI loop is very modestly better resolved, with Phe668 packed into a hydrophobic pocket and residues 669–670 drawn in slightly closer to the loop centroid. Both maps contain a pocket of density near the threefold axis of symmetry, adjacent to Pro421, Pro632, and His631, corresponding to one residue of genomic deoxynucleotide. In both structures, residues 455 through 458 (located in surface loop VR-IV) lack strong density likely due to local flexibility.

**TABLE 1:** Cryo-EM data collection, refinement, and validation statistics

	AAV7 (EMD-22412) (PDB 7JOT)
Data collection and processing	
Magnification voltage (kV)	130,000 × 200
Electron exposure (e–/Å^2^)	32
Defocus range (μm)	0.24–2.83
Pixel size (Å)	1.038
Symmetry imposed	I
Initial particle images (no.)	103,227
Final particle images (no.)	71,811
Map resolution (Å)	2.7
FSC threshold	0.143
Refinement	
Initial model used (PDB code)	6 U95
Model resolution (Å)	2.78
FSC threshold	0.5
Model composition	
Non-hydrogen atoms	4,131
Protein residues	518
Mean B factor (Å^2^)	34
R.m.s. deviations	
Bond lengths (Å)	0.0102
Bond angles (°)	1.12
Validation	
MolProbity score	2.03
Clashscore	6.35
Poor rotamers (%)	3.98
Ramachandran plot	
Favored (%)	96.51
Allowed (%)	3.49
Disallowed (%)	0

AAVrh.10 belongs to clade E and is the closest capsid in terms of amino acid identity (88.4%) to AAV7 whose structure is deposited online (PDBID: 6V1G). RMSD between these two structures is 0.7 Å (Fig. 3A). Primary backbone differences lie in VR-I (RMSD 1.85 Å), a loop previously implicated in conferring the ability to cross the blood-brain barrier for AAVrh.10 (23). The protein backbone was modeled confidently within the calculated map in this area, meaning these differences are not due to a high degree of disorder or flexibility. Structural alignment of AAVrh.10 and closely related, non-blood-brain barrier-penetrating capsid AAV8 shows perfect overlay of the Cα backbone between identical VR-I sequences (Fig. 3B). Interestingly, AAV7 does not cross the blood-brain barrier (see below), and its VR-I loop adopts a completely different conformation. The conserved Ser268 residue (AAV7 numbering) shared by several blood-brain barrier crossing capsids, including AAV9 and AAVrh.10, is present in AAV7, but residues at the base of the VR-IV loop in proximity to the conserved VR-I serine are more similar to AAV8 (24). Taken together, this suggests that the ability to cross the blood-brain barrier is driven by determinants in both VR-I and VR-IV acting in combination and mobilized during structural changes during glycan and protein receptor binding (25 – 27).

### Biodistribution

**Fig 4:**
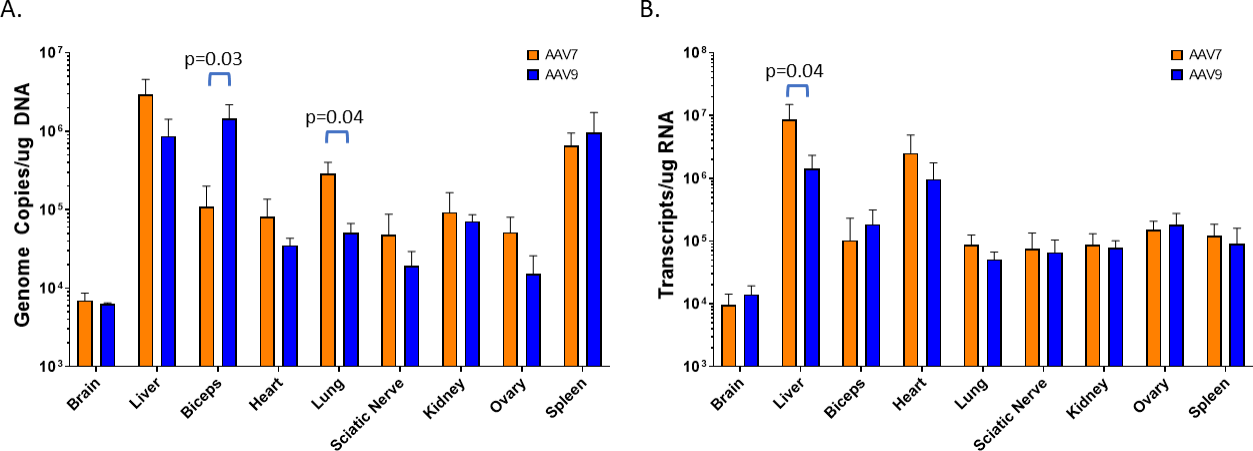
Biodistribution of AAV7 and AAV9 in female C57BL/6 mice after IV administration (*n* = 5) of 1 × 1013 GC/kg. Average AAV genome copy number over total DNA input (**A**) and transgene transcript (EGFP) copy number over input RNA (**B**) as measured by quantitative polymerase chain reaction (qPCR). Error bars represent standard deviation.

To characterize AAV7 in direct comparison with a widely used capsid, AAV7 and AAV9 vectors with EGFP transgenes were administered intravenously into 6–8-week-old C57BL/6 mice (*n* = 5) at 1 × 10^13^ GC/kg. Vector genome copy analysis indicates that many of the sampled tissues were transduced similarly by both AAV7 and AAV9, including kidney, ovary, and spleen (Fig. 4A). Interestingly and unexpectedly, AAV7 genomes and transcript were detected in the mouse brain at levels equivalent to AAV9, suggesting that AAV7 may cross the blood-brain barrier (Fig. 4B). Lung transduction was superior with AAV7 vector, showing a fivefold increase over AAV9 for the presence of vector DNA (*P* = 0.04). Likewise, skeletal muscle transduction (represented by bicep tissue) was 13 times higher in mice dosed with AAV9 (vector DNA, *P* = 0.03). Though both lung and skeletal muscle transduction trends were present when examined by transgene transcriptional level (RNA), the differences were diminished and not significant. Only liver EGFP expression of AAV7-transduced mice was significantly different than with AAV9 (sixfold increase; *P* = 0.04). Cardiac tissues showed increased AAV7 transduction and transcription over AAV9, but the increase was not statistically significant. To further differentiate AAV7 as a unique capsid, we sought a more direct and comprehensive method to characterize transgene expression through direct visualization of vector-induced protein expression in a whole-mouse model.

**Fig 5:**
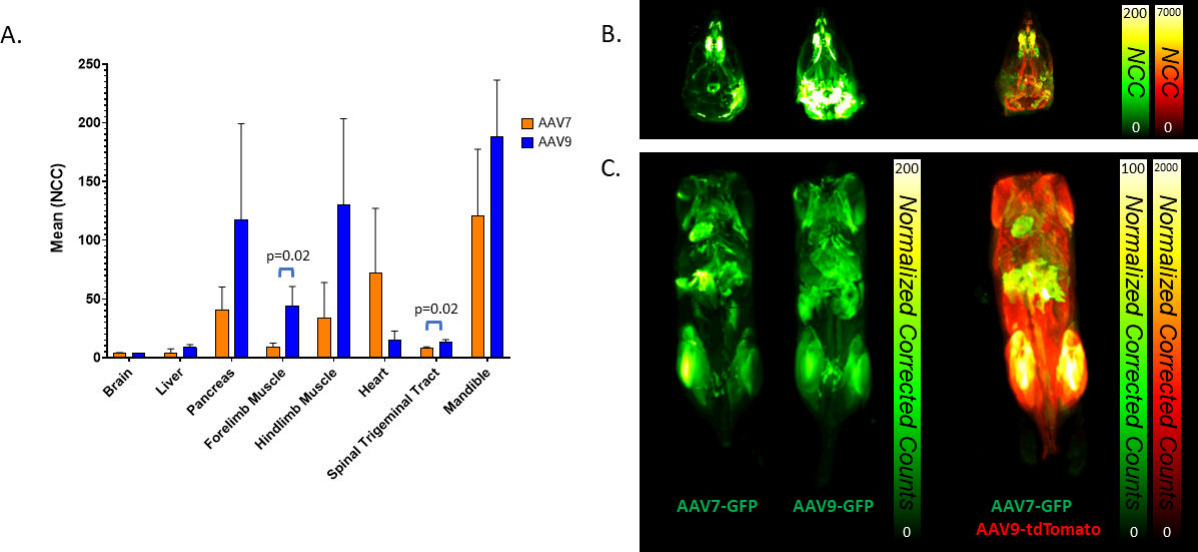
CFT analysis of C57BL/6 mouse tissues 3 weeks after IV administration of 2 × 1014 GC/kg AAV7 and AAV9 carrying a CAG.EGFP transgene (*n* = 3). (**A**) Quantitative normalized corrected count (NCC) was determined by measurement within fixed volumes placed in organs. (**B**) Representative reconstructed maximum intensity projections of animals administered single vectors (left) or both AAV7.CAG.EGFP and AAV9.CAG.tdTomato (right).

Cryofluorescence tomography (CFT) of whole mice was performed using an Xerra imager (Emit Imaging) 3 weeks after IV administration of AAV7 or AAV9 carrying an EGFP transgene or co-administration of AAV7.CAG.EGFP and AAV9.CAG.tdTomato (2 × 10^14^ GC/kg). AAV-administered animals were imaged together with non-administered animals, under identical imaging settings, to control for autofluorescence. Control animals showed no fluorescent signal in the examined organs, compared to AAV animals, when visualized on the same scale. In the animals receiving single vector administrations, EGFP expression in kidney, lung, and spleen was examined, but undetectable above background levels. Liver fluorescence was not statistically significantly different between AAV7 and AAV9 (Fig. 5A). We hypothesize the difference in liver compared to the previous biodistribution study may be due to the increase in dose, which was necessary to obtain strong enough fluorescent protein expression to visualize without immunohistochemistry (IHC), and an additional PCR-based biodistribution study was performed (*n* = 2) to support CFT findings at the higher dose (see Fig. S1 in the supplemental material). CFT supports the biodistribution findings in the brain, showing the presence of weak fluorescence in the brain for both AAV7 and AAV9, though not significantly different from each other. In the spinal trigeminal tract, AAV9 transduction was significantly higher (*P* = 0.02) than AAV7 by 1.5×, which may indicate a preference of AAV9 for targeting trigeminal ganglion cells and by extension dorsal root ganglion (DRG). Skeletal muscle (forelimb and hindlimb) analysis further emphasizes increased expression by transduction with AAV9. In forelimb, AAV9-treated animals expressed EGFP 4.9-times more than AAV7 (*P* = 0.02). In contrast, heart transduction by AAV7 over AAV9 was observed (Fig. 5A, C, 6A and B). These differences are best visualized in the dual-vector administration animals, where expression within the same animal is represented by AAV7 in green and AAV9 in red (Fig. 5B and C). Note that due to the difference in fluorescence intensity between fluorophores, this group is not meant to make a direct comparison between AAV7 and AAV9; however, in the context of the single vector EGFP groups, it does aid in visualization of locationalization of AAV7 for heart and AAV9 for skeletal muscle. Additional videos of whole-body CFT can be found in Fig. S2 in the supplemental material.

**Fig 6:**
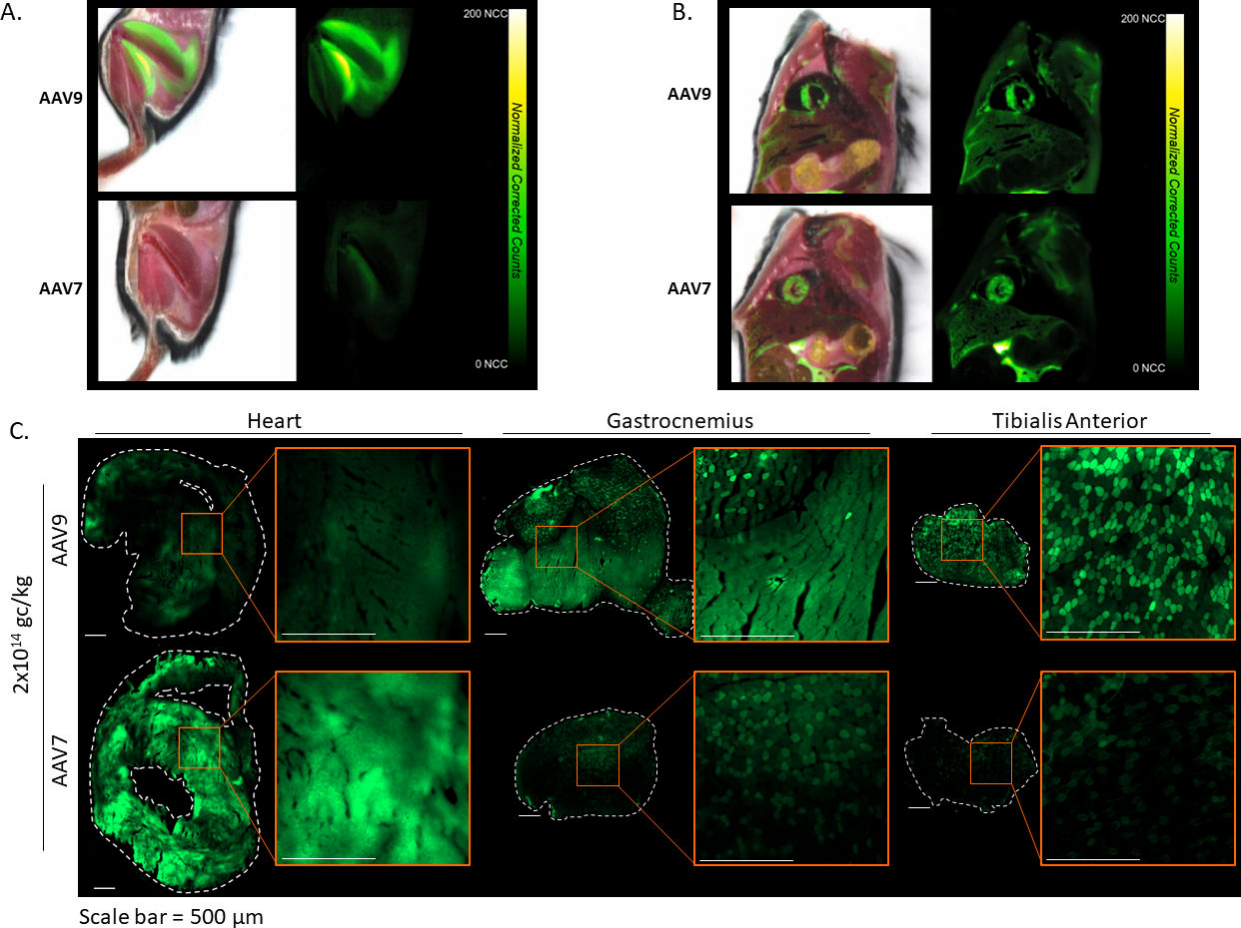
Muscle transduction by AAV9 (top) and AAV7 (bottom). Representative CFT images of EGFP fluorescence (right) in hind limb muscle (**A**) and heart (**B**) overlaid on white light images (left). (**C**) Additional histological study of native EGFP fluorescence in the skeletal and cardiac muscle after IV administration of AAV9 (top) and AAV7 (bottom) (2 × 1014 GC/kg). Whole section images (10×) of heart (left), gastrocnemius (middle), and tibialis anterior (right). Dotted lines represent the tissue edge, and zoomed images are shown in the orange inset boxes. Scale bar = 200 µm

A surprising finding from CFT was the presence of fluorescence in facial structures, some of it highly concentrated. As this is not typically an area sampled by traditional biodistribution methods, this finding has been previously overlooked, although other bony regions (e.g., femur) have been examined by others (28, 29). In particular, the mandible showed the highest intensity of EGFP fluorescence of any other tissue quantified for both AAV7 and AAV9 (Fig. 5A and B). As the mouse mandible is surrounded by muscle tissue that likely was transduced, we conducted an additional study to further ascertain detailed information about the transduction seen in and around mouse facial structures and investigate tropism differences between AAV7 and AAV9.

**Fig 7:**
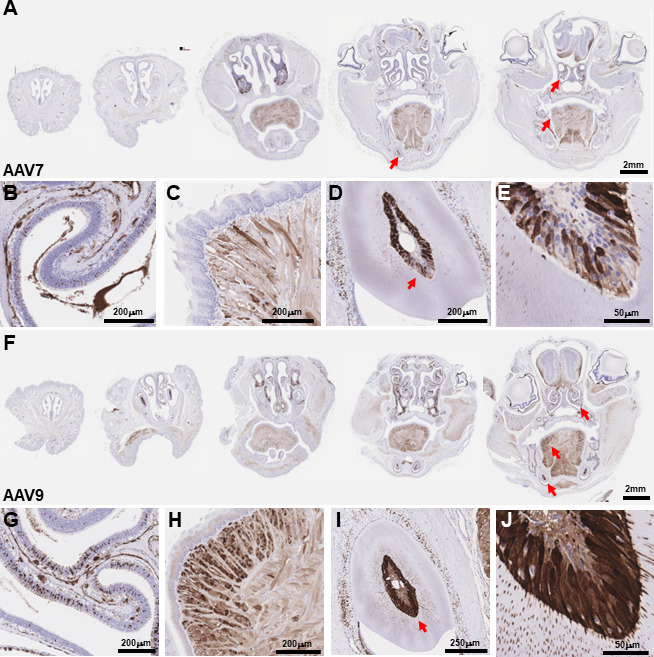
EGFP expression in the face following IV administration of AAV7 (**A**) and AAV9 (**F**) (2 × 1014 GC/kg). Red arrows indicate locations of higher magnification images. Expression in the face was widespread including strong transduction of olfactory epithelium (**B, G**), muscle cells in the tongue (**C, H**), and odontoblasts in the incisor root (**D–E, I–J**).

Immunofluorescence or immunohistochemistry was performed on mouse tissues after IV administration of AAV7 or AAV9 carrying an EGFP transgene (3 weeks post-dosing; 2 × 10^14^ GC/kg). Transgene expression in the heart and skeletal muscle (gastrocnemius and tibialis anterior) confirmed our previous observation of AAV7 transduction superiority in heart and AAV9 in the skeletal muscle (Fig. 6C). Examination of the liver, lung, and kidney reiterated similar transduction between AAV7 and AAV9, although expression of the transgene in the liver of animals treated with AAV9 was more widespread and uniform (Fig. S3A and D). Expression in the lung was present in lymphoid tissue and alveola for both vectors (Fig. S3B and E). Expression in the kidney was observed only weakly in the cortex and medulla but strongly in the membrane tissue surrounding the kidney (Fig. S3C and F). Interestingly, by histological evaluation of the decalcified mouse head, we were able to observe widespread transduction by both AAV7 and AAV9 in the muscle cells of the tongue, olfactory epithelium, lacrimal and Harderian glands beneath the eye, and odontoblasts in the incisor root (Fig. 7), which confirms our CFT results of vector transduction of the boney structures of the face, especially the mandible.

**Fig 8:**
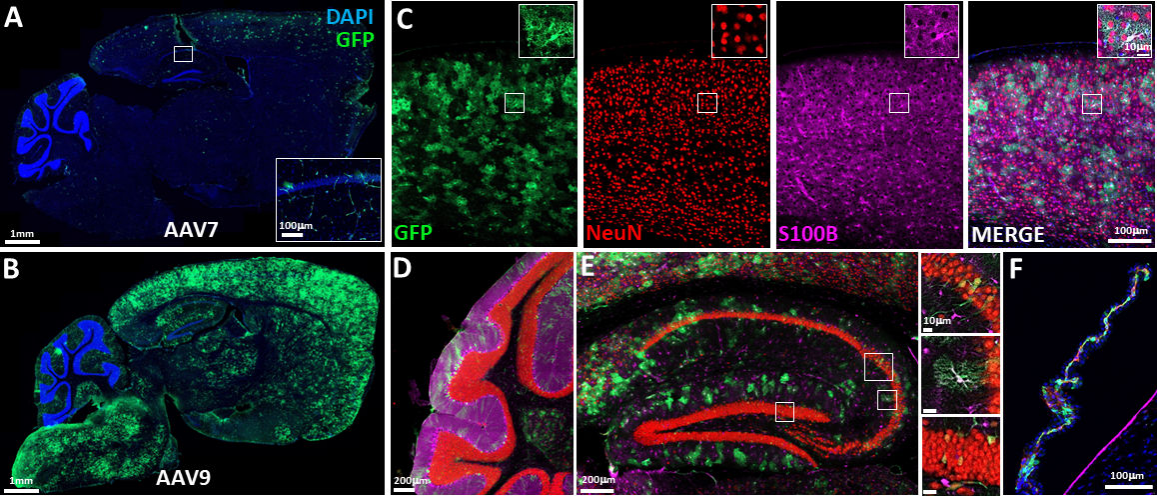
Tiled confocal images of EGFP expression (amplified by IHC) in the CNS following IV administration of AAV7 (**A**) and AAV9 (**B–F**) (2 × 1014 GC/kg). (**A**) EGFP + cells were observed scattered throughout the cortex and were predominantly observed to be astrocytes. Sparser labeling was observed in the striatum, thalamus, brainstem, and cerebellum. As shown in the inset, additional EGFP expression was observed in blood vessels of the hippocampus. (**B**) Dense expression of EGFP was observed throughout the entire brain. (**C**) Higher magnification imaging of EGFP expression in the cortex from the AAV9 representative example shown in (**B**). Multi-plexed IHC for EGFP, NeuN, and S100B demonstrates AAV9 transduction almost exclusively in the astrocytes in the cortex. (**D**) Cerebellum with EGFP expression observed in Purkinje neurons. (**E**) Hippocampus, showing EGFP expression following AAV9 administration in astrocytes, CA2 pyramidal neurons, and dentate gyrus granule cells. (**F**) Choroid plexus in the lateral ventricle.

Confocal imaging of EGFP expression (amplified by IHC) in the mouse brain after IV administration of AAV7-EGFP showed positive cells, predominantly astrocytes, scattered throughout the cortex. Only sparse transduction was observed in the striatum, thalamus, brainstem, and cerebellum (Fig. 8A). EGFP expression was primarily localized to blood vessels of the hippocampus, which was different from our observations in the AAV9-treated animals where there was dense expression of EGFP throughout the entire brain (Fig. 8B through F). IHC for EGFP, NeuN, and S100B demonstrates AAV9 transduction of exclusively astrocytes in the cortex (Fig. 8C) (30). EGFP expression in cerebellum was observed in Purkinje neurons (Fig. 8D). In the hippocampus, EGFP expression following AAV9 administration was seen in both astrocytes as well as CA2 pyramidal neurons and neurons of the dentate gyrus granule cell layer (Fig. 8E). AAV9 IV administration also transduced choroid plexus in the lateral ventricle (Fig. 8F).

Overall, AAV7 and AAV9 show differences in tropism and transduction strength in several key tissues. AAV7 could be a potential candidate for heart-directed indications, while AAV9 remains superior for skeletal muscle and brain transduction via blood-brain barrier crossing. Although qPCR-based biodistribution methods indicated that AAV7 transduces the brain, we showed via imaging study that AAV7 remains limited to the vascular endothelial cells of blood vessels, even at higher doses, whereas AAV9 was able to robustly transduce the brain from an IV administration, as previously described in literature (30). Both vectors may be viable options for the treatment of disorders involving bone, sinus tissues, and salivary or lacrimal glands.

Overall, AAV7 is a potent vector both *in vitro* and *in vivo* and is a viable candidate for clinical use in gene therapy. AAV7 vectors can be produced at titers on par with clinically used AAV8 and AAV9. Our results suggest that AAV7 does interact with AAVR, and AAVR overexpression improves *in vitro* transduction, which could be useful for the development of potency assays. Although a specific glycan receptor has yet to be identified for AAV7, our results show a potential interaction with fucosylated human milk oligosaccharides; however, these could be non-specific, as fucosylated human milk oligosaccharides are known antiviral molecules. It is possible that other attachment factors may have a larger impact on AAV7 tropism than glycans; AAV9 galactose binding residues are largely responsible for the tropism of AAV9; perhaps that is not the case for AAV7 (31, 32). Further study of glycan-AAV interaction is necessary on a per-serotype basis to determine the contributions of glycan receptors on AAV tropism. Ultimately, a less glycan-driven AAV serotype may be preferable for gene therapy, as tissue glycan profiles can be highly variable depending on several factors including species, age, and disease state (33, 34).

Where AAV7 and AAV9 differ most prominently in biodistribution is skeletal and cardiac muscle, with AAV9 being an overall stronger transducer in the skeletal muscle, and AAV7 in the heart. Both serotypes are detected in the brain via traditional biodistribution study after IV administration, and one would assume they transduce the brain with equal efficiency; however, upon closer inspection with histology/imaging, AAV7 is localized to blood vessels within the brain, whereas AAV9 transduction is widespread in astrocytes and some neurons. Surprisingly, both AAV7 and AAV9 excelled at transduction of facial bone structures and sinus tissues. This is not typically an area sampled in PCR-based biodistribution studies and supports the utility of CFT for whole-body imaging and direct visualization of transgene product.

## MATERIALS AND METHODS

### Small scale vector production

HEK293 cells were seeded 1 day before transfection. Triple plasmid DNA transfections (rep/cap, *cis*, and helper) were done with PEIpro (Polypus transfection) using standard methods. For small-scale, 10 mL suspension cultures, cells were spun down and supernatant containing vector was harvested 5 days post-transfection and stored at −80°C. Packaged vector from adherent HEK293s was harvested via freeze–thawing and purified via iodixanol gradient (35). The final product was resuspended in phosphate-buffered saline (PBS) and 0.001% Pluronic F-68. Vector was subjected to DNase I treatment to remove residual plasmid or cellular DNA and then heat treated to inactivate DNase I and denature capsids before titer determination via droplet digital PCR (ddPCR; BioRad, Hercules, CA, USA) using primers against the rabbit β-globin polyadenylation signal as described previously (36).

### Mouse study vector production

Vector preparations used for animal studies were generated at REGENXBIO vector core. Briefly, suspension HEK293 cells were triple transfected with the helper plasmid, AAV7 (NCBI: YP_077178.1) or AAV9 (Genbank: AAS99264.1) capsid plasmid, and the transgene plasmid containing CAG-EGFP or CAG-TdTomato and AAV2 ITRs. The packaged vectors were purified from clarified cell culture fluid using iodixanol gradient ultracentrifugation (37) and formulated in PBS with 0.001% Pluronic F-68. Product titer was measured using ddPCR with the same method as described for small-scale vector.

### *In vitro* transduction

HEK293T or HEK293T-AAVR cells were seeded at 2 × 10^4^ cells/well in a 96-well plate and transduced with iodixanol-purified, CAG.TdTomato-expressing vector 5 hours after seeding at 1.25 × 10^10^ vector genome copies (GC; approximately an MOI of 6.25 × 10^5^). Images were taken on a Cytation 5 Cell Imaging Multi-Mode Reader (Biotek) 48 hours after transduction with a tetramethylrhodamine (TRITC) filter cube and quantified using total fluorescence intensity.

### Glycan binding assay

Glycan Array 300 assay was performed by Raybiotech with AAV7 and AAV9 (control). Arrayed chips printed with N-glycans, glycolipid glycans, human milk oligosaccharides, tandem epitopes, and others were incubated with AAV7 and AAV9. Vector was detected via biotinylated ADK8 (for AAV7) or ADK9 (for AAV9) antibodies (PROGEN) and labeled streptavidin. Intensity was then read and analyzed.

### Cryo-electron microscopy

Amorphous carbon film was deposited on cleaved grade V1 mica (Ted Pella, Redding, California, USA) by E-beam deposition using an ACE600 (Leica Microsystems GmbH). Carbon was floated on glow-discharged Quantifoil *R2*/1 grids (Quantifoil Micro Tools GmbH) within an hour of vitrification. A volume of 3.5 µL of particles diluted in PBS was applied to the grid and plunge-frozen using a Vitrobot Mark IV (FEI Company) with no. 595 blotting paper (Ted Pella). Grids were imaged on a Talos Arctica cryo-electron microscope at 1.038 Å/pixel with a total dose of 31.92 electrons/Å^2^ over 30 frames. Images were recorded with a K2 Summit detector (Gatan) in counting mode. Movies were processed using RELION version 3.1-beta and refined to 2.7 Å resolution with icosahedral symmetry using phase-randomized AAVhu.37 as an initial model. Seventy-one thousand eight hundred eleven particles were used in the final structure out of 103,227 particles boxed. Refinement of the same data set using cryoSPARC gave an essentially identical map to refinement in RELION. Modeling was initialized by mutating the deposited model of AAVhu.37 (PDB: 6U95) to match the AAV7 sequence. Rounds of automatic refinement in Phenix and manual refinement in Coot were conducted and the final coordinates were evaluated by eye and using MolProbity. Structural figures were generated using PyMOL (v.2.1.0; Schrodinger).

### C57BL/6 mouse biodistribution

AAV7.CAG.EGFP and AAV9.CAG.EGFP vectors were injected intravenously into 6–8 weeks old, female C57BL/6 mice at a dose of 1 × 10^13^ GC/kg body weight with five mice per treatment group. The animals were euthanized and tissue samples were collected 18 days post-injection. gDNA was isolated from tissue samples using DNeasy Blood and Tissue kit (Qiagen). The gDNA samples were analyzed by qPCR using primer-probe combination specific to the EGFP sequence to determine the vector copy number in each organ. RNA was isolated from tissue samples using RNeasy Fibrous Tissue kit (Qiagen), and cDNA was produced via High-Capacity cDNA Reverse Transcription kit (Applied Biosystems). cDNA was analyzed via ddPCR using primer-probe combination specific to the EGFP sequence to determine the total transcripts per RNA input.

### Cryofluorescence tomography

#### Dosing

Female C57Bl/6 mice aged 6–8 weeks (Charles River Laboratories) were acclimated for 48 hours prior to AAV administration and divided into five groups (*n* = 3 per group): non-treated, AAV7.CAG.EGFP, AAV9.CAG.EGFP, AAV9.CAG.TdTomato, and an equimolar mixture of AAV7.CAG.EGFP and AAV9.CAG.TdTomato. Single AAV vectors were administered intravenously via tail vein, with a 200 µL bolus injection at 2 × 10^14^ GC/kg. The dual AAV group was administered 200 µL AAV7.CAG.EGFP and 200 µL AAV9.CAG.TdTomato. Animals were fed alfalfa-free chow to minimize gut auto-fluorescence during image acquisition. Animals were euthanized 3 weeks post-administration and immediately frozen whole via submersion in a dry ice-hexane bath. Frozen animals were stored at −80°C until image acquisition.

#### Image acquisition

Once frozen, mice were decapitated to allow imaging the heads in a smaller field of view to optimize resolution. Head-only samples (*n* = 2 per block) or body-only samples (*n* = 5 per block) were blocked in optimal cutting temperature (OCT) compound with fiducials to aid in image alignment and reconstruction. Sequential sectioning (25 µm for head block, 50 µm for body block) and image acquisition cycles (block-face imaging) were performed throughout the entire samples. White light and fluorescent images were acquired at each plane with autoexposure setting (EGFP channel EX470nm, EM 511/20 nm; TdTomato channel EX555nm; EM 585/11 nm).

#### Image reconstruction and analysis

Following the completion of image acquisition, planar images were reconstructed and processed to generate maximum intensity projections (MIPs) of fluorescent images and flythrough movies of both fluorescent and white light images, to represent the biodistribution of the fluorescent biomarkers. CFT images were aligned to account for movements of the camera and cryo-sectioning by identifying fiducial markers in a characteristic image using normalized cross- correlation in white light images. Background subtraction was performed by subtracting the mean signal in a user-defined region within the OCT which was considered to have zero fluorescence. Each fluorescent image was normalized to its corresponding exposure time to generate normalized corrected counts (NCCs). The images were subsampled and divided into separate images, one for each subject in the block. The aligned and separated white light images were then written into a MetalImage (MHD) format to allow for visualization using VivoQuant and generation of MIPs and flythroughs. Image acquisition and reconstruction resulted in 18.3 µm^3^ voxel size for head images and 57.4 µm^3^ voxel size for body images. Representative mean fluorescent intensity within fixed volumes, placed in selected organs, was determined to allow quantitative comparison of biomarker expression in various organs. Mean fluorescent intensity in the brain, kidneys, and heart were determined using organ phantoms.

### Histological assessment of vector expression

AAV7.CAG.EGFP and AAV9.CAG.EGFP vectors were injected intravenously (200 mL) into 6–8 weeks old, female C57BL/6 mice (Jackson Laboratory) at doses of 1 × 10^13^ GC/kg and 2 × 10^14^ GC/kg body weight with two mice per treatment group. Twenty-one days after injection, animals were transcardially perfused with cold PBS until exsanguination blanched the liver. Following perfusion, the brain, liver, skeletal muscle (tibialis anterior, gastrocnemius, and diaphragm), heart, lungs, kidney, ovary, spleen, and sciatic nerve were harvested. For lung and kidney, the left organ was flash frozen for biodistribution, whereas the right was drop-fixed in 4% paraformaldehyde (PFA) for histology. The liver was treated similarly with two lobes for biodistribution and one for histology. Liver, kidney, and lung tissue for histology were fixed in 4% PFA overnight, followed by 24 hours of 30% sucrose and then rinsed, dehydrated through graded alcohols, cleared in xylene, and infiltrated with paraffin, before being embedded in a paraffin block and sectioned at 5 mm on a cryostat. The whole heart was frozen in isopentane and later processed as described below. The brain was removed from the skull and processed as described below. The remainder of the head was kept intact and fixed in 4% PFA overnight, then switched to 30% sucrose, de-calcified with either formic acid and sodium citrate or multiple changes of EDTA for 3 weeks before being rinsed, dehydrated through graded alcohols, cleared in xylene, and infiltrated with paraffin, embedded in a paraffin block, and finally sectioned at 5 mm on a cryostat.

Hearts and skeletal muscle (gastrocnemius and tibialis anterior) were flash frozen in an isopentane/liquid nitrogen double bath. The heart was then sectioned from the base until the appearance of the ventricles and 10 µm transverse cryosections were mounted on glass slides (Superfrost, Fisherbrand). For skeletal muscle, 10 µm transverse cryosections were taken from the proximal side of the muscle and mounted on glass slides. Immediately after sectioning, sections were mounted with Permount (Electron Microscopy Sciences) and cover-slipped. Slides were cured overnight and protected from light until imaging. The next day, whole tissue sections were acquired on a Zeiss LSM 900 scanning confocal microscope operating in widefield mode with a 10× Plan-Neofluor 0.3NA objective using the tiling/stitching functions in Zeiss Zen Blue software.

The brain was hemi-sected, and the left hemisphere was flash frozen for biodistribution, while the right hemisphere was fixed in 4% PFA overnight and then cryo-protected in 30% sucrose before sectioning in the sagittal plane at 50 mm on a Leica freezing microtome and stored in a cryo-protectant solution (30% ethylene glycol and 25% glycerol in phosphate buffer). For immunohistochemistry, sections were washed in Tris buffered saline (TBS) (3 × 15 min) and then blocked in TBS++ solution, which consists of TBS, 0.25% Triton-X100, and 3% normal horse serum (Vector Laboratories, Newark, CA, USA). Sections were then incubated in TBS++ with primary antibodies for 48 hours at 4°C. The following primary antibodies were used: chicken anti-GFP (#NB100-1614, Novus Biologicals, Centennial, CO, USA) at 1:1000, mouse anti-NeuN (#ab104224, Abcam, Cambridge, MA, USA) at 1:1000, and rabbit anti-S100β (#ab52642, Abcam, Cambridge, MA, USA) at 1:1000. Subsequently, sections were washed in TBS (2 × 15 min), TBS++ for 30 min to block, and then incubated in TBS++ with secondary antibodies at 23°C for 2 hours. The following secondary antibodies were used at a 1:250 dilution: donkey anti-chicken IgG Alexa Fluor 488 (Invitrogen, Waltham, MA, USA), donkey anti-mouse IgG Alexa Fluor 555 (Invitrogen, Waltham, MA, USA), and donkey anti-rabbit IgG Alexa Fluor 647 (Invitrogen, Waltham, MA, USA). Sections were next stained with DAPI (Invitrogen, Waltham, MA, USA) at 5 mg/mL for 5 min before final washes in TBS (3 × 15 min). After IHC, sections were float mounted on glass slides, dried, and cover-slipped with mounting media (ProLong Gold anti-fade). Confocal images were obtained using a Zeiss LSM 900 Airyscan 2 using 10× and 20× objectives with tile-imaging and z-stacks that are presented as maximum projections. Tiles were stitched and brightness/contrast were adjusted in Zeiss’ Zen software.

For liver, kidney, lung, and coronal sections of the face, immunohistochemistry was performed with the thinly sectioned tissue on glass slides. To visualize EGFP expression, formalin-fixed, paraffin-embedded (FFPE) slides were deparaffinized in graded alcohols and brought to water. The slides were then retrieved with either a pH six buffer (soft tissues) or a pH nine buffer (bone). After retrieval, slides were blocked with a peroxidase block, incubated with the anti-GFP primary (ab183734, 1:300), and detected with an HRP-conjugated goat-anti-rabbit secondary. The HRP was visualized with 3,3'-diaminobenzidine (DAB). The slides were then counterstained with hematoxylin, dehydrated with graded alcohols, cleared in xylene, and mounted with a permanent mounting medium. Additionally, sections were stained for hematoxylin and eosin (H&E). As before, FFPE slides were deparaffinized with xylene, and then hydrated through graded alcohols up to water. Slides were then stained with Carazzi’s hematoxylin, washed in tap water, and placed in 95% ethanol. Slides were then counterstained with eosin-phloxine, dehydrated through graded alcohols, cleared in xylene, and coverslipped using permount as mounting media. H&E and DAB processed slides were imaged on a Leica AT2 at a resolution of 0.25 µm/px.

## Data Availability

For the AAV7 structure, cryo-electron microscopy data for AAV7 have been deposited and are available at the PDB (PDBID: 7JOT) and EMDB (EMD-22412)

## SUPPLEMENTAL MATERIAL

Supplemental figures and legends jvi.01163-23-s0001.pdf S1: Additional biodistribution of the high dose study; S2: Additional CFT videos (separate files); S3: Additional histology of liver, lung, and kidney.

Fig. S2A (video) jvi.01163-23-s0002.mp4 Using the fiducial markers embedded in the block, planar fluorescent images were aligned and reconstructed into a 3D image, visualizing the AAV-mediated expression of the fluorescent biomarkers across the entire head. One representative animal per group is shown. The reconstructed samples are presented as maximum intensity projections (MIP). GFP is scaled at 0-200 NCC, tdTomato is scaled at 0-7000 NCC.

Fig. S2B (video) jvi.01163-23-s0003.mp4 Using the fiducial markers embedded in the block, planar fluorescent images were aligned and reconstructed into a 3D image, visualizing the AAV-mediated expression of the fluorescent biomarkers across the entire body. One representative animal per group is shown. The reconstructed samples are presented as maximum intensity projections (MIP). GFP is scaled at 0-200 NCC, tdTomato is scaled at 0-7000 NCC.

Fig. S2C (video) jvi.01163-23-s0004.mp4 Planar white light and fluorescent images were co-registered to generate a movie flythrough throughout the head providing anatomical context to allow accurate identification of organs expressing GFP from one representative animal dosed with AAV7.GFP. GFP is scaled at 0-200 NCC.

Fig. S2D (video) jvi.01163-23-s0005.mp4 Planar white light and fluorescent images were co-registered to generate a movie flythrough throughout the entire animal providing anatomical context to allow accurate identification of organs expressing GFP from one representative animal dosed with AAV7.GFP. GFP is scaled at 0-200 NCC.

Fig. S2E (video) jvi.01163-23-s0006.mp4 Planar white light and fluorescent images were co-registered to generate a movie flythrough throughout the head providing anatomical context to allow accurate identification of organs expressing GFP from one representative animal dosed with AAV9.GFP. GFP is scaled at 0-200 NCC.

Fig. S2F (video) jvi.01163-23-s0007.mp4 Planar white light and fluorescent images were co-registered to generate a movie flythrough throughout the entire animal providing anatomical context to allow accurate identification of organs expressing GFP from one representative animal dosed with AAV9.GFP. GFP is scaled at 0-200 NCC.

Fig. S2G (video) jvi.01163-23-s0008.mp4 Planar white light and fluorescent images were co-registered to generate a movie flythrough throughout the head providing anatomical context to allow accurate identification of organs expressing GFP from one representative animal dosed with AAV7.GFP+AAV9.TdTomato.

Fig. S2H (video) jvi.01163-23-s0009.mp4 Planar white light and fluorescent images were co-registered to generate a movie flythrough throughout the entire animal providing anatomical context to allow accurate identification of organs expressing GFP from one representative animal dosed with AAV7.GFP+AAV9.TdTomato.

Fig. S2I (video) jvi.01163-23-s0010.mp4 Planar white light and fluorescent images were co-registered to generate a movie flythrough throughout the head providing anatomical context to allow accurate identification of organs expressing tdTomato from one representative animal dosed with AAV7.GFP+AAV9.TdTomato. tdTomato is scaled at 0-7000 NCC.

Fig. S2J (video) jvi.01163-23-s0011.mp4 Planar white light and fluorescent images were co-registered to generate a movie flythrough throughout the entire animal providing anatomical context to allow accurate identification of organs expressing tdTomato from one representative animal dosed with AAV7.GFP+AAV9.TdTomato. tdTomato is scaled at 0-7000 NCC.

ASM does not own the copyrights to Supplemental Material that may be linked to, or accessed through, an article. The authors have granted ASM a non-exclusive, world-wide license to publish the Supplemental Material files. Please contact the corresponding author directly for reuse.

## References

[1] Mueller C , Flotte TR . 2008. Clinical gene therapy using recombinant adeno-associated virus vectors. Gene Ther 15:858–863. doi:10.1038/gt.2008.68 18418415

[2] Daya S , Berns KI . 2008. Gene therapy using adeno-associated virus vectors. Clin Microbiol Rev 21:583–593. doi:10.1128/CMR.00008-08 18854481 PMC2570152

[3] Mingozzi F , High KA . 2011. Therapeutic in vivo gene transfer for genetic disease using AAV: progress and challenges. Nat Rev Genet 12:341–355. doi:10.1038/nrg2988 21499295

[4] Knipe DM , Howley PM. 2013. Fields virology, 6th ed. Wolters Kluwer/Lippincott Williams & Wilkins Health, Philadelphia, PA.

[5] Varnavski AN , Zhang Y , Schnell M , Tazelaar J , Louboutin J-P , Yu Q-C , Bagg A , Gao G , Wilson JM . 2002. Preexisting immunity to adenovirus in rhesus monkeys fails to prevent vector-induced toxicity. J Virol 76:5711–5719. doi:10.1128/jvi.76.11.5711-5719.2002 11991999 PMC137042

[6] Wu Z , Asokan A , Samulski RJ . 2006. Adeno-associated virus serotypes: vector toolkit for human gene therapy. Mol Ther 14:316–327. doi:10.1016/j.ymthe.2006.05.009 16824801

[7] Gao G , Vandenberghe LH , Alvira MR , Lu Y , Calcedo R , Zhou X , Wilson JM . 2004. Clades of adeno-associated viruses are widely disseminated in human tissues. J Virol 78:6381–6388. doi:10.1128/JVI.78.12.6381-6388.2004 15163731 PMC416542

[8] Gao GP , Alvira MR , Wang L , Calcedo R , Johnston J , Wilson JM . 2002. Novel adeno-associated viruses from rhesus monkeys as vectors for human gene therapy. Proc Natl Acad Sci U S A 99:11854–11859. doi:10.1073/pnas.182412299 12192090 PMC129358

[9] Samaranch L , Salegio EA , San Sebastian W , Kells AP , Bringas JR , Forsayeth J , Bankiewicz KS . 2013. Strong cortical and spinal cord transduction after AAV7 and AAV9 delivery into the cerebrospinal fluid of nonhuman primates. Human Gene Therapy 24:526–532. doi:10.1089/hum.2013.005 23517473 PMC3655626

[10] Gray SJ , Foti SB , Schwartz JW , Bachaboina L , Taylor-Blake B , Coleman J , Ehlers MD , Zylka MJ , McCown TJ , Samulski RJ . 2011. Optimizing promoters for recombinant adeno-associated virus-mediated gene expression in the peripheral and central nervous system using self-complementary vectors. Hum Gene Ther 22:1143–1153. doi:10.1089/hum.2010.245 21476867 PMC3177952

[11] Shao W , Pei X , Cui C , Askew C , Dobbins A , Chen X , Abajas YL , Gerber DA , Samulski RJ , Nichols TC , Li C . 2019. Superior human hepatocyte transduction with adeno-associated virus vector serotype 7. Gene Ther 26:504–514. doi:10.1038/s41434-019-0104-5 31570819 PMC6923567

[12] Lock M , Alvira M , Vandenberghe LH , Samanta A , Toelen J , Debyser Z , Wilson JM . 2010. Rapid, simple, and versatile manufacturing of recombinant adeno-associated viral vectors at scale. Hum Gene Ther 21:1259–1271. doi:10.1089/hum.2010.055 20497038 PMC2957274

[13] Pillay S , Meyer NL , Puschnik AS , Davulcu O , Diep J , Ishikawa Y , Jae LT , Wosen JE , Nagamine CM , Chapman MS , Carette JE . 2016. An essential receptor for adeno-associated virus infection. Nature 539:108–112. doi:10.1038/nature19835 PMC4962915 26814968

[14] Meisen WH , Nejad ZB , Hardy M , Zhao H , Oliverio O , Wang S , Hale C , Ollmann MM , Collins PJ . 2020. Pooled screens identify GPR108 and TM9SF2 as host cell factors critical for AAV transduction. Mol Ther Methods Clin Dev 17:601–611. doi:10.1016/j.omtm.2020.03.012 32280726 PMC7139131

[15] McDougald DS , Duong TT , Palozola KC , Marsh A , Papp TE , Mills JA , Zhou S , Bennett J . 2019. CRISPR activation enhances in vitro potency of AAV vectors driven by tissue-specific promoters. Mol Ther Methods Clin Dev 13:380–389. doi:10.1016/j.omtm.2019.03.004 31024980 PMC6477656

[16] Zhang R , Xu G , Cao L , Sun Z , He Y , Cui M , Sun Y , Li S , Li H , Qin L , Hu M , Yuan Z , Rao Z , Ding W , Rao Z , Lou Z . 2019. Divergent engagements between adeno-associated viruses with their cellular receptor AAVR. Nat Commun 10:3760. doi:10.1038/s41467-019-11668-x 31434885 PMC6704107

[17] Zhang R , Cao L , Cui M , Sun Z , Hu M , Zhang R , Stuart W , Zhao X , Yang Z , Li X , Sun Y , Li S , Ding W , Lou Z , Rao Z . 2019. Adeno-associated virus 2 bound to its cellular receptor AAVR. Nat Microbiol 4:675–682. doi:10.1038/s41564-018-0356-7 30742069

[18] Xu G , Zhang R , Li H , Yin K , Ma X , Lou Z . 2022. Structural basis for the neurotropic AAV9 and the engineered AAVPHP.eB recognition with cellular receptors. Mol Ther Methods Clin Dev 26:52–60. doi:10.1016/j.omtm.2022.05.009 35755945 PMC9198364

[19] Mietzsch M , Broecker F , Reinhardt A , Seeberger PH , Heilbronn R . 2014. Differential adeno-associated virus serotype-specific interaction patterns with synthetic heparins and other glycans. J Virol 88:2991–3003. doi:10.1128/JVI.03371-13 24371066 PMC3958061

[20] Shen S , Bryant KD , Brown SM , Randell SH , Asokan A . 2011. Terminal N-linked galactose is the primary receptor for adeno-associated virus 9. J Biol Chem 286:13532–13540. doi:10.1074/jbc.M110.210922 21330365 PMC3075699

[21] Bode L . 2012. Human milk oligosaccharides: every baby needs a sugar mama. Glycobiology 22:1147–1162. doi:10.1093/glycob/cws074 22513036 PMC3406618

[22] Mietzsch M , Jose A , Chipman P , Bhattacharya N , Daneshparvar N , McKenna R , Agbandje-McKenna M . 2021. Completion of the AAV structural atlas: serotype capsid structures reveals clade-specific features. Viruses 13:101. doi:10.3390/v13010101 33450892 PMC7828300

[23] Albright BH , Storey CM , Murlidharan G , Castellanos Rivera RM , Berry GE , Madigan VJ , Asokan A . 2018. Mapping the structural determinants required for AAVrh.10 transport across the blood-brain barrier. Mol Ther 26:510–523. doi:10.1016/j.ymthe.2017.10.017 29175157 PMC5835146

[24] Mietzsch M , Barnes C , Hull JA , Chipman P , Xie J , Bhattacharya N , Sousa D , McKenna R , Gao G , Agbandje-McKenna M . 2020. Comparative analysis of the capsid structures of AAVrh.10, AAVrh.39, and AAV8. J Virol 94:e01769-19. doi:10.1128/JVI.01769-19 31826994 PMC7158714

[25] Penzes JJ , Chipman P , Bhattacharya N , Zeher A , Huang R , McKenna R , Agbandje-McKenna M . 2021. Adeno-associated virus 9 structural rearrangements induced by endosomal trafficking pH and glycan attachment. J Virol 95:e0084321. doi:10.1128/JVI.00843-21 34260280 PMC8428384

[26] Meyer NL , Hu G , Davulcu O , Xie Q , Noble AJ , Yoshioka C , Gingerich DS , Trzynka A , David L , Stagg SM , Chapman MS . 2019. Structure of the gene therapy vector, adeno-associated virus with its cell receptor, AAVR. Elife 8:e44707. doi:10.7554/eLife.44707 31115336 PMC6561701

[27] Kaelber JT , Yost SA , Webber KA , Firlar E , Liu Y , Danos O , Mercer AC . 2020. Structure of the AAVhu.37 capsid by cryoelectron microscopy. Acta Crystallogr F Struct Biol Commun 76:58–64. doi:10.1107/S2053230X20000308 32039886 PMC7010358

[28] Yang Y-S , Xie J , Wang D , Kim J-M , Tai PWL , Gravallese E , Gao G , Shim J-H . 2019. Bone-targeting AAV-mediated silencing of Schnurri-3 prevents bone loss in osteoporosis. Nat Commun 10:2958. doi:10.1038/s41467-019-10809-6 31273195 PMC6609711

[29] Yang YS , Xie J , Chaugule S , Wang D , Kim JM , Kim J , Tai PWL , Seo SK , Gravallese E , Gao G , Shim JH . 2020. Bone-targeting AAV-mediated gene silencing in osteoclasts for osteoporosis therapy. Mol Ther Methods Clin Dev 17:922–935. doi:10.1016/j.omtm.2020.04.010 32405514 PMC7210389

[30] Foust KD , Nurre E , Montgomery CL , Hernandez A , Chan CM , Kaspar BK . 2009. Intravascular AAV9 preferentially targets neonatal neurons and adult astrocytes. Nat Biotechnol 27:59–65. doi:10.1038/nbt.1515 19098898 PMC2895694

[31] Pulicherla N , Shen S , Yadav S , Debbink K , Govindasamy L , Agbandje-McKenna M , Asokan A . 2011. Engineering liver-detargeted AAV9 vectors for cardiac and musculoskeletal gene transfer. Mol Ther 19:1070–1078. doi:10.1038/mt.2011.22 21364538 PMC3129791

[32] Bell CL , Gurda BL , Van Vliet K , Agbandje-McKenna M , Wilson JM . 2012. Identification of the galactose binding domain of the adeno-associated virus serotype 9 capsid. J Virol 86:7326–7333. doi:10.1128/JVI.00448-12 22514350 PMC3416318

[33] Tena J , Lebrilla CB . 2021. Glycomic profiling and the mammalian brain. Proc Natl Acad Sci U S A 118:e2022238118. doi:10.1073/pnas.2022238118 33380465 PMC7817126

[34] Lee J , Ha S , Kim M , Kim SW , Yun J , Ozcan S , Hwang H , Ji IJ , Yin D , Webster MJ , Shannon Weickert C , Kim JH , Yoo JS , Grimm R , Bahn S , Shin HS , An HJ . 2020. Spatial and temporal diversity of glycome expression in mammalian brain. Proc Natl Acad Sci U S A 117:28743–28753. doi:10.1073/pnas.2014207117 33139572 PMC7682437

[35] Zolotukhin S , Byrne BJ , Mason E , Zolotukhin I , Potter M , Chesnut K , Summerford C , Samulski RJ , Muzyczka N . 1999. Recombinant adeno-associated virus purification using novel methods improves infectious titer and yield. Gene Ther 6:973–985. doi:10.1038/sj.gt.3300938 10455399

[36] Lock M , Alvira MR , Chen SJ , Wilson JM . 2014. Absolute determination of single-stranded and self-complementary adeno-associated viral vector genome titers by droplet digital PCR. Hum Gene Ther Methods 25:115–125. doi:10.1089/hgtb.2013.131 24328707 PMC3991984

[37] Crosson SM , Dib P , Smith JK , Zolotukhin S . 2018. Helper-free production of laboratory grade AAV and purification by iodixanol density gradient centrifugation. Mol Ther Methods Clin Dev 10:1–7. doi:10.1016/j.omtm.2018.05.001 30073177 PMC6069679

