# Supplementary material for: Characterization and biodistribution of under-employed gene therapy vector AAV7
Source: J Virol. 2023 Oct 16;97(11):e01163-23. doi: 10.1128/jvi.01163-23 (PMC10688378; doi:10.1128/jvi.01163-23)
Supplement: Supplemental figures and legends — S1: Additional biodistribution of the high dose study; S2: Additional CFT videos (separate files); S3: Additional histology of liver, lung, and kidney. [file jvi.01163-23-s0001.pdf]

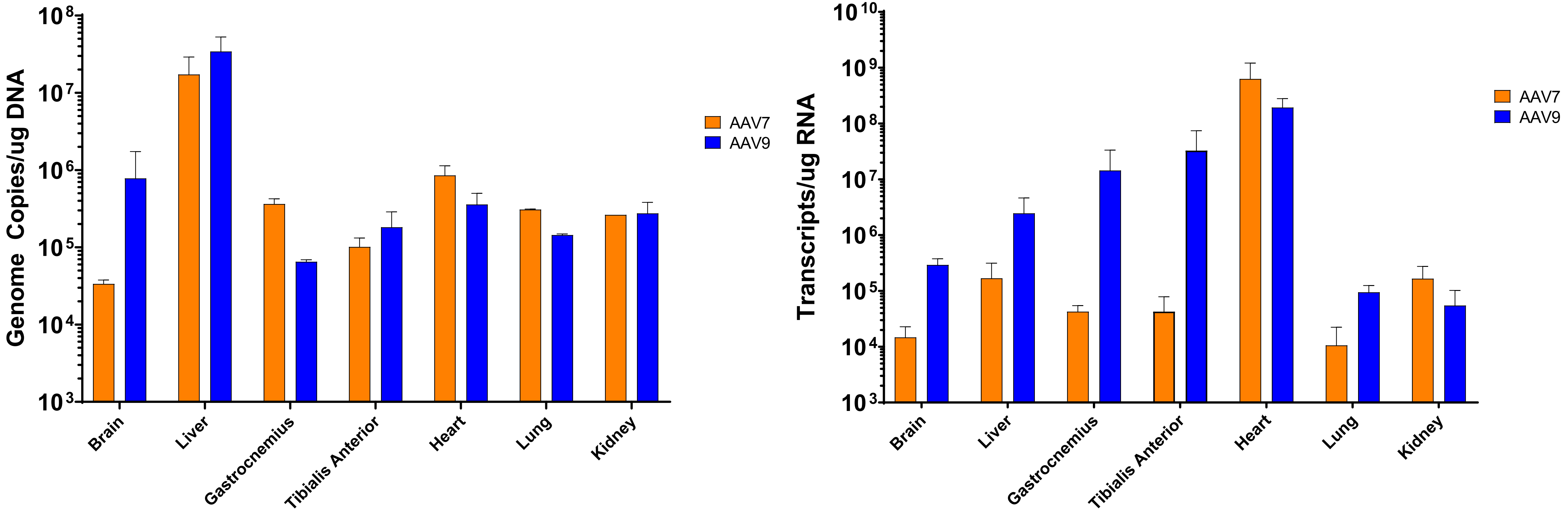

Supplementary figure 1. Biodistribution of AAV7 and AAV9 in C57BL/6 mice after IV administration (n=2) of $2x10^{14}$ gc/kg. Average AAV genome copy number over total DNA input (A) and transgene transcript (EGFP) copy number over input RNA (B) as measured by ddPCR. Error bars represent standard deviation.

See video files

Supplementary figure 2. (A-B) Using the fiducial markers embedded in the block, planar fluorescent images were aligned and reconstructed into a 3D image, visualizing the AAV-mediated expression of the fluorescent biomarkers across the entire head (A) or body (B). One representative animal per group is shown. The reconstructed samples are presented as a maximum intensity projections (MIP). GFP is scaled at 0-200 NCC, tdTomato is scaled at 0-7000 NCC. (C-J) Planar white light and fluorescent images were co-registered to generate a movie flythrough throughout the entire animal (D, F, H, and J) or head (C, E, G, and I), providing anatomical context to allow accurate identification of organs expressing GFP or tdTomato. One representative animal is shown per group: AAV7.GFP (C,D), AAV9.GFP (E,F), AAV7.GFP+AAV9.TdTom (G, H, I, J)

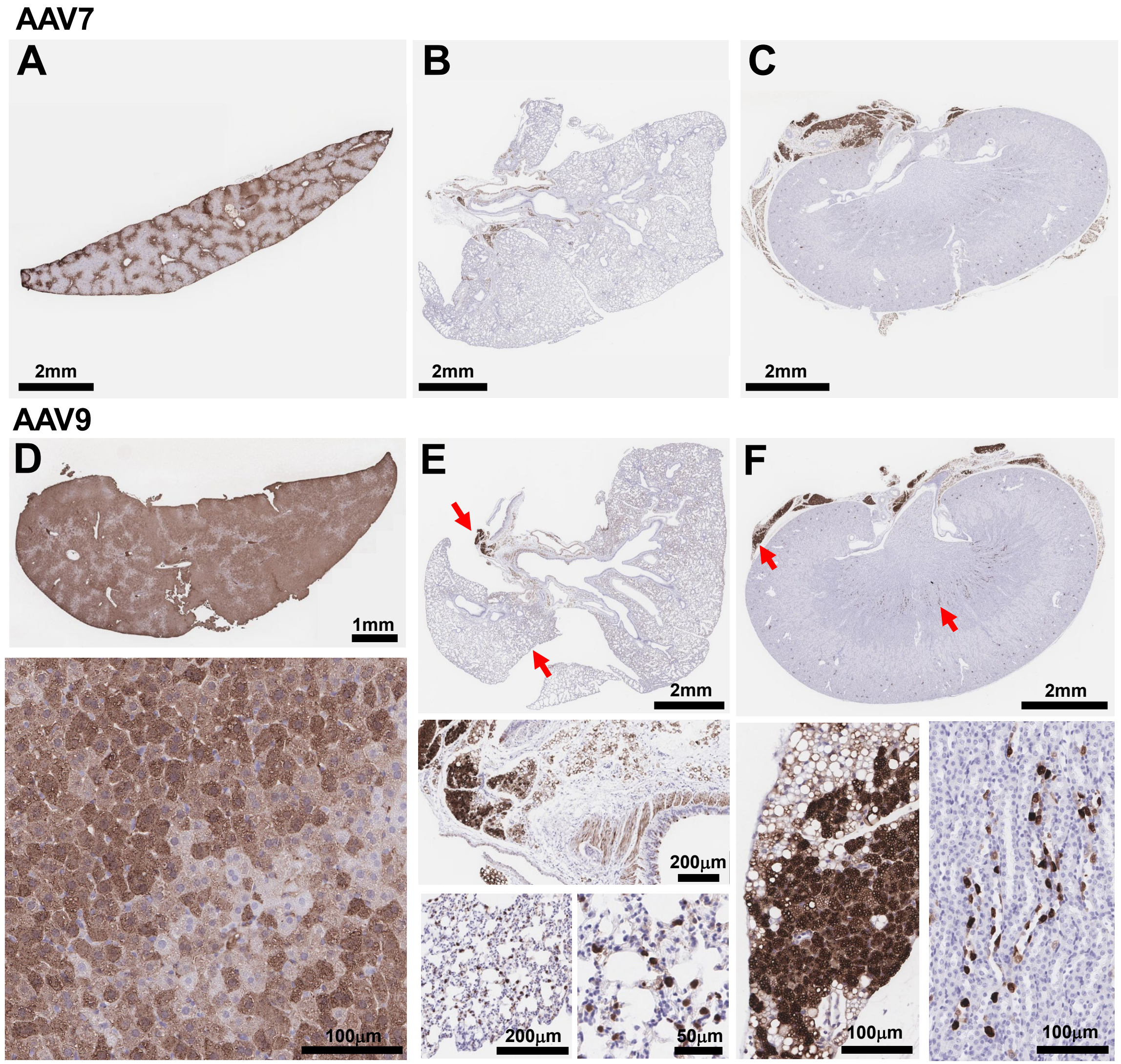

Supplementary figure 3. EGFP expression in the periphery following IV administration of AAV7 (A-C) and AAV9 (D-F) in the liver (A and D), lung (B and E), and kidney (C and F) ($2x10^{14}$ gc/kg). Red arrows indicate locations of higher magnification images.
